# Mean Platelet Volume: is it an Emerging Marker or an Exaggeration?

**DOI:** 10.21470/1678-9741-2018-0425

**Published:** 2019

**Authors:** Ugur Kaya, Yavuzer Koza, Abdurrahim Colak

**Affiliations:** 1 Department of Cardiovascular Surgery, Ataturk University, Faculty of Medicine, Erzurum, Turkey.; 2 Department of Cardiology, Ataturk University, Faculty of Medicine, Erzurum, Turkey.; 3 Department of Cardiovascular Surgery, Ataturk University, Faculty of Medicine, Erzurum, Turkey.

Dear Editor,

We thank Dr. Coban^[1]^ for his extremely valuable comments and criticisms on our recently published article^[2]^ in Brazilian Journal of Cardiovascular Surgery. Firstly, our study had a retrospective nature, and, therefore, we think that seasonal changes in mean platelet volume (MPV) levels may be considered in prospectively designed studies. In our study, the MPV values were analyzed with aperture-impedance technology using the Beckman Coulter Gen-S (*Beckman Coulter* Corporation, Miami, USA; *GEN-S)* device and the blood samples were obtained by ethylenediaminetetraacetic acid (EDTA). We agree with the various techniques of different instruments for measuring the complete blood count can lead to variable MPV results. In a study^[3]^, the MPV measurements varied up to 17.8% by the instruments. The results of another study revealed that MPV can be measured accurately by both methods of anticoagulation; EDTA and citrate if analysis were performed within 1h of sampling^[4]^. In our study, the same standardized blood tubes were used for sampling and all blood samples were analyzed within 1h after venipuncture.

Secondly, there are conflicting results about the effect of the anticoagulant used and the time between blood collection and measurement on MPV^[5]^. Therefore, these concerns still need a standardization. Studies which are planning prospectively about MPV will provide the standardization of anticoagulant used and the time between blood collection, and measurement because of data accuracy and reliability.

Lastly, we could not understand an experimental study^[6]^ example which criticizes a retrospectively designed human study. Furthermore, in that study Histidine-Tryptophan-Ketoglutarate (HTK) cardioplegia was compared to DelNido cardioplegia and those authors investigated the cardioprotective effects of the newly developed HTK cardioplegia, not MPV. Although no one can avoid criticism, it is important to emphasize that the animal models may not accurately mimic human disease.
